# Supplementary material for: Assessing vegetation cover and valuing ecosystem services in southwestern Ethiopia: Implications for conservation
Source: PLoS One. 2024 Jun 21;19(6):e0304456. doi: 10.1371/journal.pone.0304456 (PMC11192345; doi:10.1371/journal.pone.0304456)
Supplement: Attachment — Submitted filename: rebuttal letter.docx [file pone.0304456.s001.docx]

**Mizan ATVET College**

<http://www.mizan-atvet.edu.et>

[z.ageru@yahoo.com](mailto:z.ageru@yahoo.com) Mobile: +251914346360

| Date: | 16-Apr-24 |
| --- | --- |

PONE-D-24-00098

Assessing Vegetation Cover and Valuing Ecosystem Services in Southwestern Ethiopia: Implications for Conservation.

Dear Editor,

Thank you for considering our manuscript. After careful consideration, we accept all the comments forwarded by all reviewers and we respond some explanations and corrections after each reviewers comment as follows.

Respond to reviewers:

Reviewer #1: 1.Abstract
This part briefly introduces the whole process of the study, and clearly states the results and views of the study. Although the research is very valuable, the background and purpose of the research are not stated. Nowadays, there are many scientific problems that need to be solved in the world or in Ethiopia. However, I do not know from the abstract what kind of international environment the author conducts the research based on, what kind of problems to deal with and what kind of purpose he started it. It is recommended to start this section with a 1-2 sentence brief explanation.
It is not accurate to use the expression "biodiversity" in the first sentence of the abstract. Firstly, biodiversity includes the diversity of animals and plants, and this paper does not explore the diversity of animals. Secondly, this paper discusses the economic value of different vegetation cover and ecosystem services, but does not mention the role or relationship of plant diversity characteristics, and does not involve the calculation and analysis of plant diversity indicators of different vegetation cover types. Therefore, it is suggested that the author reconsider the expression method.

Based on the comments Provided, we have added the international environment “ the area is parts Eastern Afromontane Biodiversity Hotspot” and the purpose also stated, final we have changed the word biodiversity

2. Introduction
This section explains the reasons for studying the vegetation cover of Ethiopia and the value of its ecosystem services. A series of anthropogenic factors, such as urban expansion and deforestation, have resulted in the degradation and loss of forests in the region. Ethiopia's rich flora and fauna have been damaged to varying extent. In this process, the monitoring system based on satellite remote sensing plays a great role, and this study is also based on this. So at present, have some scholars conducted research on this level? What are the latest research results in this field in the world? Do their results provide the basis for this study? And what does this study do on the basis of previous studies? The authors are requested to supplement the content of the review in appropriate places.

We have put references about land cover of the world and some recent findings of parts of the study area and similar environment in adjacent areas.

3. Methodology
In the part of the Study site, I have clearly understood the basic information of Bechi-Sheko in Ethiopia. Please briefly explain why you choose this region instead of other regions.
In the part of Data Analysis, I have been clear about the research method and data processing process, but why do we use this method in this study? What are the advantages of similar methods? Were the same types of methods compared and screened before this study began?

We have stated why we choose the area and why the method is preference “ During data analysing, different Supervised Classification classifiers include CART, RandomForest, NaiveBayes and SVM was used, however, A random forest classifier trained on this dataset yielded best accuracy results for different vegetation types.”

4. Result
This part fully introduces the research results and the process of drawing production, and provides necessary explanations and explanations for the drawing. However, the significance of the two indicators of overall accuracy and kappa coefficient is widely known. It is suggested to properly check the description of the Result part and simplify the explanation of the redundant part. Avoid over-description.
The image of Figure4.5 is not high in definition, please provide a clear enough image of the result.

Based on the comment we have corrected the image in accordance with plos one requirement and rewrite some redundant parts

5. Discussion
The first paragraph of this part should be the content of the Result part, please merge and adjust.

Corrected

In the third paragraph of this section, the view that "wetland classification has been neglected" is confusing. Due to differences in techniques and classification criteria, different studies produce different classifications, which are equal. In your study, the result that wetlands occupy 4.35% of the study area only shows that the classification of wetlands can be obtained using your method and criteria, and its ecosystem service value is correspondingly very limited. Since the area occupies a small proportion and the contribution is limited, how can we prove that the humidity area is essential? In addition, MODIS and ESA cover the whole world, and the scope of this study is small, so I doubt this conclusion, please further explain.

Based on the comment, we put further explanation to avoid misunderstanding and to clear the potentials of world land cover and their limitation as well. The wetland coverage in the study area is not faily distributed in all parts of the study area, most of wetland cover found in shay bench districts of Bench Sheko, therefore 4.35% is big enough in such case. The study emphases vegetative wetlands may confuse with grasslands during classification.

In the fourth paragraph of this part, since the choice of evaluation method will affect the result, it is necessary to explain the reasons for the choice of method and the choice of region. Please adjust it in accordance with the issues in section "3. Methodology".

Corrected

Reviewer #2: The study has great potential and is very important especially given the area where the study was carried out. Despite this, the study needs major revisions especially in terms of structure and writing. Not respecting the guidelines makes it difficult to give precise indications because the number of lines and/or the number of pages are missing, however, for example, the paragraph "Data Analysis" is difficult to read and understand with some points where it appears to be simple annotations (i.e. "Radiometric conversions and correction."). Not making all acronyms explicit makes reading difficult. In the results, the NDVI value is mentioned but not shown.

Based on the comment, we have done many editorial problems, for example Reference style, line spaces . we rewrite the data analysis grammar problem part.

The discussions also seem quite messy. I propose a discussion for each service analysed in relation to the type of land cover. In the conclusions highlight more the importance and maybe give some suggestions in terms of conservation.
The English should be revised as well as the spelling part. Sentences should always begin with a capital letter.

The discussion is rewritten based on the comments.
